# A functional micro-electrode mapping of ventral thalamus in essential tremor

**DOI:** 10.1093/brain/awy192

**Published:** 2018-07-23

**Authors:** David J Pedrosa, Peter Brown, Hayriye Cagnan, Veerle Visser-Vandewalle, Jochen Wirths, Lars Timmermann, John-Stuart Brittain

**Affiliations:** 1 Department of Neurology, University Hospital of Marburg and Gießen, Marburg, Germany; 2 Nuffield Department of Clinical Neurosciences and MRC Brain Network Dynamics Unit, University of Oxford, Oxford, UK; 3 Department of Psychiatry, University Hospital of Marburg and Gießen, Marburg, Germany; 4 Department of Stereotactic and Functional Neurosurgery, University Hospital Cologne, Cologne, Germany; 5 Department of Neurology, University Hospital Cologne, Cologne, Germany; 6 School of Psychology, University of Birmingham, Birmingham, UK

**Keywords:** essential tremor, ventrolateral thalamus, posterior subthalamic area, electrophysiology

## Abstract

Deep brain stimulation enables the delivery of therapeutic interventions to otherwise inaccessible areas of the brain while, at the same time, offering the unique opportunity to record from these same regions in awake patients. The posterior ventrolateral thalamus has become a reliable deep brain stimulation target for medically-refractory patients suffering from essential tremor. However, the contribution of the thalamus in essential tremor, and even whether posterior ventrolateral thalamus is the optimal target, remains a matter of ongoing debate. There are several lines of evidence supporting clusters of activity within the posterior ventrolateral thalamus that are important for tremor emergence. In this study we sought to map the functional properties of these clusters through microelectrode recordings during deep brain stimulation surgery. Data were obtained from 10 severely affected patients with essential tremor (12 hemispheres) undergoing deep brain stimulation surgery. Our results demonstrate power and coherence maxima located in the inferior posterior ventrolateral thalamus and immediate ventral region. Moreover, we identified distinct yet overlapping clusters of predominantly efferent (driving) and afferent (feedback) activity, with a preference for more efferent contributors, consistent with a net role in the driving of tremor output. Finally, we demonstrate that resolvable thalamic spiking activity directly relates to background activity and that the strength of tremor may be dictated by phase relationships between efferent and afferent pockets in the posterior ventrolateral thalamus. Taken together, these results provide important evidence for the role of the inferior posterior ventrolateral thalamus and its border region in essential tremor pathophysiology. Such results progress our mechanistic understanding and promote the adoption of next-generation therapies such as high resolution segregated deep brain stimulation electrodes.

## Teaser

Using microelectrode recordings in patients with essential tremor undergoing DBS surgery, Pedrosa *et al*. demonstrate that the ventrolateral thalamus shows maximum tremor-related activity and the highest coherence with electromyographic activity in the contralateral arm. Efferent pockets of activity outnumber afferent, and the phase relationship between these components modulates tremor amplitude.

## Introduction

Essential tremor refractory to medical treatment is a frequent indication for deep brain stimulation (DBS) lead placement. Compelling evidence has shown dramatic tremor relief with remarkable improvement in quality of life due to thalamic high frequency stimulation (Benabid *et al.*, 1991; Pahwa *et al.*, 2006). DBS surgery also offers the opportunity to record brain activity that may provide important insight into the pathomechanisms of essential tremor.

A growing body of evidence indicates a relationship between thalamic activity and tremulous muscles. The posterior ventrolateral thalamus (VLp) exhibits increased coherence with the EMG at individual tremor frequency (Marsden *et al.*, 2000; Kane *et al.*, 2009; Pedrosa *et al.*, 2014*b*) with coherence displaying a somatotopic organization (Pedrosa *et al.*, 2012). Furthermore, beta activity in the motor thalamus inversely correlates with tremor amplitude (Basha *et al.*, 2014), corroborating the pivotal role of the thalamus in essential tremor. However, the local field potential activity most commonly studied represents the superposition of activity from a large number of neighbouring neural sources, and this lack of spatial discrimination is an important limitation. To appreciate the contribution and organization of thalamic neurons to tremor, a more detailed view is required. This is especially important when we consider that there is ongoing debate over the efficiency of stimulating the thalamus, versus stimulation applied immediately below its VLp division (King *et al.*, 2017).

Understanding the pathomechanisms of essential tremor with higher spatial and temporal precision is of important clinical relevance. While the efficiency of DBS has been extensively demonstrated, side effects may oppose satisfactory tremor relief. A more thorough insight into the functional anatomy may promote the use of next-generation DBS systems such as directional leads (Tinkhauser *et al.*, 2018) or ultrasound-based thalamotomy (Elias *et al.*, 2016). A detailed knowledge of the timing of tremor-related activity could also promote the development of biomarkers to support closed-loop forms of neurostimulation (Cagnan *et al.*, 2017).

Previous studies recording thalamic microelectrode data have reaffirmed several important concepts about tremor generation. Hence, tremor-related activity is most prominent in cerebellar recipient subdivisions of the ventrolateral thalamus, that is in Jones’ nomenclature, the VLp (for a review see Hamani *et al.*, 2006). There, Hua *et al.* (1998) also demonstrated tonic firing related to the EMG of both agonists and antagonists resulting in oscillatory activity. Moreover, thalamic spiking activity appears to precede activity seen in the EMG that underlies postural or intention tremors (Zakaria *et al.*, 2013). Interestingly, there is evidence of segregated pathways within the thalamus (Pedrosa *et al.*, 2012) with different cells mediating efferent drive and afferent feedback (Lenz *et al.*, 1994). In view of the possible segregated efferent drive and resonant reafference, a more detailed view on the characteristics of tremor in both the thalamus and the areas immediately ventral are important in the development of tailored therapies for essential tremor.

To enhance our knowledge of the interaction between thalamic areas and those in the immediate vicinity, we investigated microelectrode recordings obtained during DBS lead placement from 10 patients (12 hemispheres). During each surgery, up to five trajectories of microelectrodes were acquired simultaneously whilst mapping the ventrolateral thalamus and surrounding areas. Up to 10 recording levels were obtained per trajectory. Additionally, we analysed the predominant afferent (feedback) and efferent (driving) contribution of these signals along with their spiking activity. In-so-doing we tested the over-arching hypothesis that tremor-related activity in focal clusters of thalamic neurons organizes and helps drive tremulous arm movements.

## Materials and methods

The study was approved by the local Ethics committee and carried out in accordance with the Declaration of Helsinki. All patients gave informed written consent prior to participating.

### Surgical procedure and electrophysiological recordings

Ten patients suffering from essential tremor and receiving surgery for DBS lead implantation were enrolled. All patients showed an impairing postural tremor, which was refractory to medical treatment. Clinical data are provided in Table 1.

**Table 1:** General data on essential tremor-patients undergoing surgery

Subject	Gender	Age, years	Disease duration, years	Tremor frequency, Hz	Target MNI coordinates^a^	Trajectories	Recording levels
1	M	63.6	30	4.9	11.5, −18.3, −2.5	2	5
2	F	75.2	31	4.0	−11.9, −18.7, 0.0	3	9
3	F	71.1	25	3.8	−12.3, −18.4, −2.4	5	5
4^a^	F	73.2	15	4.5	−11.9, −20.4, −2.0/10.9 −20.3, −2.0	4/3	10/10
5	M	48.4	30	7.8	−11.5, −20.3, −1.0	4	12
6	F	54.7	20	4.8	−11.3, −21.0, −0.2	4	7
7^a^	F	67.4	45	5.8	−12.6, −15.2, 0.4/11.2, −16.3, 1.6	1/1	8/8
8	F	69.1	15	5.8	−10.5, −18.1, −4.4	4	8
9	M	61.7	29	5.0	−10.9, −18.3, −0.9	5	11
10	M	72.3	14	4.9	11.2, −21.4, −1.5	5	9
Mean ± SD	6:4	65.7 ± 8.2	25.4 ± 9.2	5.1 ± 1.1	*x*^b^ = 11.5 ± 06; *y* = − 18.9 ± 1.8; *z* = − 0.4 ± 1.9	3.4 ± 1.9	8.5 ± 2.1

^a^Compared to stereotactic coordinates, here the references is the anterior commissure instead of the mid-commisural line.

^b^Lateral coordinates were transformed to positive values, to allow comparability.

Electrodes were implanted bilaterally with all patients rescinded from medication for at least 12 h prior to surgery. The VLp and its lower border were targeted using stereotactic high resolution MRI (T_1_-weighted) and Schaltenbrand-Wahren Atlas coordinates (Schaltenbrand and Wahren, 1977). [The Schaltenbrand-Wahren atlas does not specifically address the VLp as a region but according to Krack *et al.* (2002), this region partially corresponds to Hassler’s ventral intermediate nucleus. To maintain a uniform nomenclature throughout this paper, we will in the following refer to the ventral intermediate nucleus as VLp.] The standard coordinates of the lower border of the VLp were: (i) 5.0–5.5 mm posterior to the mid-commissural point in the *y*-axis; (ii) at the level of the AC-PC line (*z*-axis); and (iii) 12–14.5 mm lateral to midline (*x*-axis). The target coordinates for each subject are displayed in Table 1. In cases where the targeted location was between the inferior VLp and the posterior subthalamic area, the corresponding coordinates for the *z*-axis were lower.

Electrode positioning was confirmed by clinical testing (tremor suppression and/or emergence of side-effects due to stimulation) as well as radiography in two planes. Intraoperative test stimulation indicated their adequate localization at the target region. The final placement of the DBS electrode (model 3387 or 3389, Medtronic Corporation) was based on a profile of maximizing tremor reduction and minimizing side effects (heaviness or weakness of the contralateral limbs, numbness of the extremities or face, or dysarthria) during intraoperative test stimulation with microelectrodes at different recording depths. For this purpose, stimulation pulses of 60 µs duration at a frequency of 130 Hz were applied at the macroelectrode ring at increasing amplitudes up to maximally 4 mA or until patients reported side-effects.

**Figure 1:**
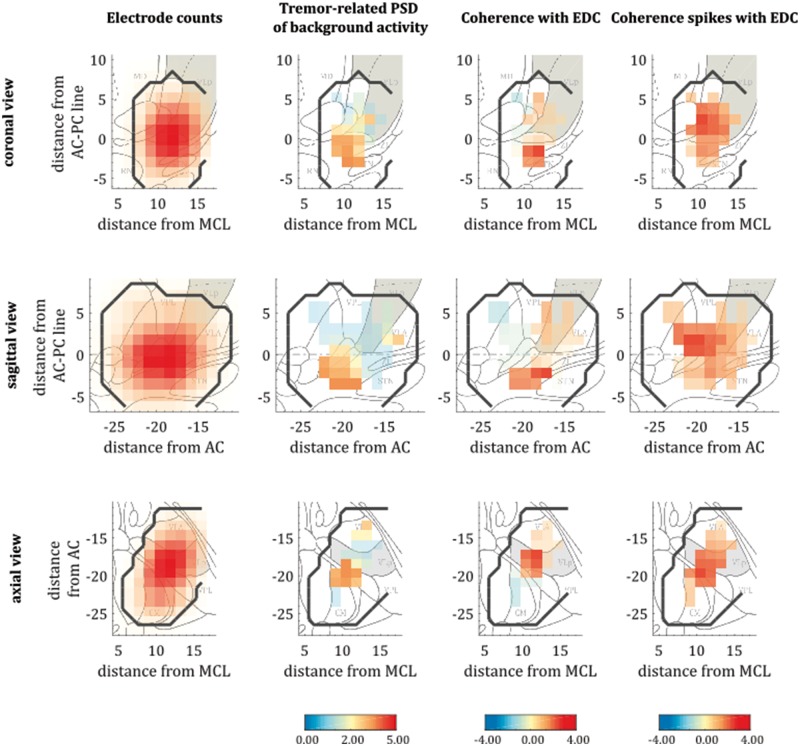
**Histograms of activity and maps of power and thalamomuscular coherence based on the Stereotactic Atlas of the Human Thalamus and Basal Ganglia (**Morel, 2007**).** *Left column*: The distribution of the recordings is illustrated in all three axes (sagittal, coronal and axial). Red colour indicates an increasing number of recordings in this area. The second column from the *left* illustrates the distribution of log-differences between power spectra during tremor and at baseline, converted into *t*-statistics. The two columns on the *right* show the normalized difference in coherence between tremor condition and baseline, again as *t*-statistics for background activity and EMG and spike-trains and EMG, respectively. The right two columns indicate increases in relative power and coherence specifically in the lower regions of the ventrolateral thalamus and/or the posterior subthalamic area. Data from the left hemispheres were flipped for comparability in the presented analysis. Black contour lines designate the sampled region as determined by the maps of electrode counts, whereas grey areas mark the respective VLp. CM* = *centromedian nucleus; MD* = *mediodorsal nucleus; RN* = *red nucleus; STN* = *subthalamic nucleus; VLA = ventral lateral anterior nucleus; VPL* = *ventral posterior lateral nucleus; ZI* = *zona incerta.

Intraoperative data were recorded using a commercially available system (INOMED MER System 2.4 beta). Sedation such as opioids, propofol or other hypnotic agents were withdrawn at least 15 min before testing. Data were obtained from up to five electrodes (central, anterior, medial, posterior and lateral; from now on termed ‘trajectories’). These electrodes consist of a macroelectrode ring (diameter 800 μm) with a high impedance microelectrode tip (diameter 4 μm) 1.5 mm below the ring. All data were recorded from the tip at a sampling frequency of 25 kHz. Time restrictions during surgery meant only one hemisphere was recorded in the majority of subjects. Moreover, some subjects presented anatomical confounds which impeded the recording of all five trajectories such as a blood vessel crossing (increased risk of bleeding), or severe artefacts within channels led to the exclusion of these trajectories (Table 1 for further details). Separate recordings were obtained over a distance of 5–12 mm, starting from 6–8 mm above the target point and ending in the areas below the thalamus, i.e. 1–4 mm beyond the planned target. We term the different recording depths as ‘levels’ from now on. For a 3D histogram of the electrode positions see Fig. 1.

Two conditions were tested in consecutive order: (i) patients resting their arms in a comfortable position (baseline); or (ii) patients elevating their forearm contralateral to the implanted hemisphere at an angle of ∼30°, spreading their fingers and maintaining this posture (tremor condition). Subjects performed both tasks for 60 s while awake and without speaking or performing any other activities. After these recordings the electrodes were lowered 1 mm and the paradigm was repeated. We simultaneously recorded activity of the extensor digitorum communis muscle of the forearm using surface EMG electrodes at a sampling frequency of 2.5 kHz.

The identification of the coordinates for the different levels and trajectories followed linear algebra. Specifically, two perpendicular planes to the known vector $c^{\to }$ between entry and target were constructed. From these, parallel vectors at a Euclidean distance of 2 mm were drawn from which the locations of each recording site were determined in MNI space. According to this, the exact determination of coordinates is impossible and we will refer to coarse anatomic localizations below.

### Data processing of background and spiking activity

All data were visually inspected for artefacts. Particularly, arm movements at the onset of the baseline ‘rest’ condition or high amplitude signals at either start or end of the thalamic recordings were identified through visual inspection of electrophysiological activity and discarded.

**Figure 3:**
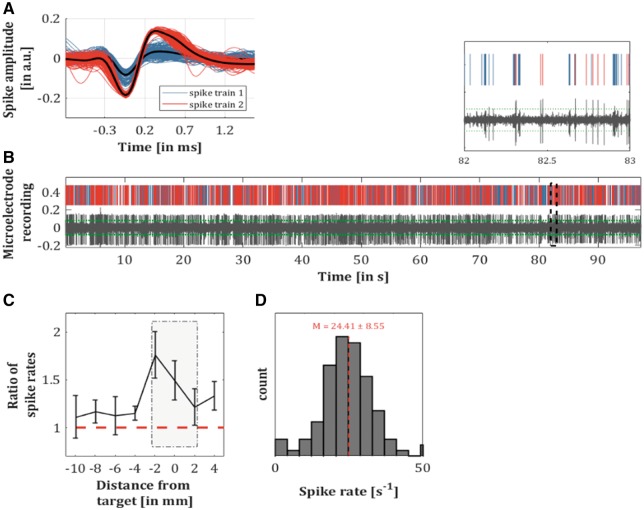
**Analyses for thalamic spiking activity.** (**A**) Illustration of spiking activity with two identified clusters. (**B**) Corresponding preprocessed recording of a microelectrode with threshold at 4σ (dotted green line) and magnified section. (**C**) Ratio of spike count during tremor and at rest for the different recording levels. Mean ratios at target ± 2 mm differed significantly from those at a further distance (*Z* = − 2.02, *P* = 0.043, *r* = − 0.41). (**D**) Spike rates during tremor for the recordings at ±2 mm from target. The dotted red line illustrates the overall average of firing rate at 24.4 spikes per second.

Preprocessing of the EMG followed standard procedures, i.e. notch filtering the line noise, high-pass filtering (30 Hz), then full-wave rectification and low-pass filtering at 50 Hz with both filters using a third-order Butterworth filter (Negro *et al.*, 2015). Microelectrode data were separated into two measurements: (i) background activity; and (ii) spiking activity. Background unit activity was isolated by high-pass filtering the local field potential at 300 Hz and rectifying the data. Spiking activity with an amplitude of more than four standard deviations (4σ) was identified and epochs of 1 ms before and 3 ms after each spike were replaced with a randomly selected non-spiking portion of the same signal. To avoid the introduction of spurious activity, the edges of these 4 ms intervals were cubic spline interpolated. The resulting signals were low-pass filtered at 300 Hz, downsampled to 500 Hz after applying an anti-aliasing filter and saved as different file. The processed data are hereafter referred to as background activity. The identified spiking activity was subjected to a clustering algorithm based on the wave characteristics (Quiroga *et al.*, 2004). After indexing the spiking activity, data were resampled at 2.5 kHz. A representative time series with corresponding spikes is shown in Fig. 3A and B.

### Spectral analyses of background activity

Power spectra of thalamic background and EMG activity and their respective cross-spectra as well as cross-spectra between spike trains and EMG were obtained using Thomson’s multitaper method with three tapers (Thomson, 1982; Mitra and Pesaran, 1999). Data were segmented into non-overlapping 3 s epochs, resulting in a frequency resolution of 0.33 Hz and the resulting spectra averaged. We denote the cross-spectrum between two signals x and y at frequency f as $S_{xy}(f)$ with individual power spectra [$S_{xx}(f)$ and $S_{yy}(f)$] analogously defined. Coherence between the preprocessed EMG signal and each recording sensor was calculated as (Halliday *et al.*, 1995):

(1) $${\left|R_{xy}\left(f\right)\right|}^{2}=\frac{{\left|\langle S_{xy}(f)\rangle \right|}^{2}}{\langle S_{xx}(f)\rangle \cdot \langle S_{yy}(f)\rangle }$$

where 〈〉 denotes the average spectra over all independent epochs. To explore the spatial representation of changes with respect to baseline, power and coherence were considered as follows: for power estimates the difference of the log of power values between tremor and baseline was computed. Tremor amplitude was calculated as the average spectral power around the peak tremor frequency ±3 Hz. Coherence was first z-transformed and then normalized to account for different number of samples according to Bokil *et al.* (2007).

(2) $$Z_{coh}(f)=\frac{tanh^{-1}\left({\left|R_{xy}^{\left(cond\right)}(f)\right|}^{2}\right)-\left(\frac{1}{\left(df-2\right)}\right)\;}{\sqrt{\frac{1}{\left(df-2\right)}}}$$

with ${\left|R_{xy}^{\left(cond\right)}(f)\right|}^{2}\;$ representing coherence values for the respective condition, $tanh^{-1}$ the inverse hyperbolic tangent and df the number of degrees of freedom (the number of epochs multiplied by the number of tapers per epoch in this case).

For the spatial mapping shown in Fig. 1, a 3D grid with 1.5 mm^3^ mesh was created and recording coordinates were assigned to their closest grid point. Histograms were mapped along with relative power and coherence changes. All metrics were smoothed per subject using a 3D Gaussian kernel with 2 mm^3^ standard deviation (SD) and then between-subjects *t*-statistics calculated. The coordinates of maximal power and coherence were compared using Hotelling’s T^2^ test for two multivariate dependent samples. Additionally, Euclidean distances between coordinates of individual power and coherence maxima and contacts used for stimulation were estimated. For this purpose, postoperative high resolution CT scans were re-imported into the planning software (STP 3.0 and STVX, Stryker Leibinger) for superimposition on preoperative MRI, resulting in stereotactic coordinates of the four contacts within one hemisphere. Finally, we considered coherence between spiking activity (with EMG) and background activity (with EMG) in a linear mixed-effects model with ‘subject’ as random-factor to test within-subject dependences of coherence on recording location (Supplementary material).

### Granger causality

**Figure 2:**
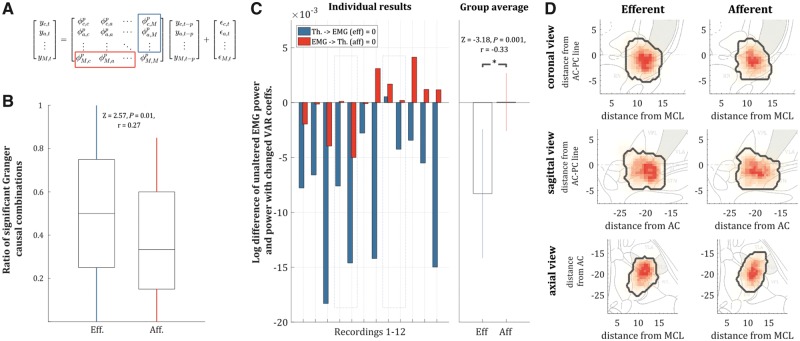
**The differentiated view on afferent and efferent connections.** (**A**) Matrix notation of the VAR model for one of the recording levels with all trajectories (c = central; a = anterior; M = extensor digitorum communis muscle); $y_{c}$ is the time series of the central electrode, $\varphi_{c,a}$ is the regression coefficient between the time series of the central and the anterior electrode, $y_{c,t-p}$ is the time series $y_{c}$ with a lag of *P* and $\varepsilon_{c,t}$ are the residuals. The red and blue rectangles illustrate the afferent and efferent coefficients with respect to the EMG activity. (**B**) Ratio of significant efferent and afferent Granger causality values for the recordings ± 2 mm from the target. The ratios were significantly different with more efferent than afferent connections (*P* = 0.01). (**C**) When selectively setting the afferent or efferent regression coefficients to zero (see red and blue rectangles in **A**) and re-estimating the EMG power resulting from this, there was a consistent reduction in tremor amplitude with respect to the unaltered system for efferent connections, but not the afferent connections. (**D**) Histogram of significant efferent and afferent pairwise Granger causality estimates. Although the afferent connections appear more widespread within a wide range of the posterior subthalamic area while efferent connections displayed a more focal localization, the geometric centre across all subjects did not differ significantly [T^2^ = 2.10, *F*(3,6) = 0.53, *P* = 0.68]. The grey areas mark the respective VLp. AC = anterior commissure; MCL = mid-commissural line.

Background activity at the target location ±2 mm and corresponding EMG signals were analysed for Granger causality in the frequency domain using the Multivariate Granger Causality (MVGC) Toolbox. For this purpose, a vector-autoregressive (VAR) model was defined for every available trajectory and recording level (see VAR model in Fig. 2A). The VAR coefficients were estimated from the cross-power spectral density of the data. This was particularly useful as it allows the estimation of Granger causality for individual tremor frequencies; for details, see Barnett and Seth (2014). To test for significance of the estimated causality measures, data derived statistics were applied. For every combination of recordings (i.e. each trajectory and simultaneously recorded EMG), the sequence of trials for one time series was shuffled and Granger causality recomputed. This was repeated 500 times per hemisphere to produce a null-distribution against which the original (unshuffled) data were assessed against the 95th percentile. Recordings were categorized as either (i) predominantly afferent (predicted by the peripheral tremor); (ii) predominantly efferent (predictive of the peripheral tremor); or (iii) neither. This was assessed by their relative proportion of efferent and afferent connectivities, as conveyed through the VAR model. We then computed the ratio of significant efferent:afferent combinations and compared these ratios at the group level using a two-sided Wilcoxon rank sum test. Hence, we use the terms afferent and efferent connectivity to distinguish thalamic activity that is better (Granger) predicted by EMG, or better predicts EMG, respectively. We acknowledge that this is an oversimplification of the complex amount of afferences integrated in the thalamus, which may disregard features of distinct somatosensory fibres or input resulting from other parts of the CNS.

To further ascertain the specific influence of efferent and afferent connections on EMG tremor amplitude, we set either the afferent connections (last column of VAR coefficients) or the efferent connections (last row of VAR coefficients) to zero, mimicking a lack of efferent drive and/or feedback in the system (for a visual illustration see Fig. 2A). We then evaluated the change induced in EMG power. The idea of this ‘virtual lesion’ on offline data is exemplified in more detail in the Supplementary material, where simulated data are also provided to reinforce the validity and interpretational value of this method. Significant differences in EMG power were compared using a two-sided Wilcoxon rank sum test. This approach mimicked the effect of removing either efferent drive to the muscles, or afferent feedback from the muscles. In a final step, we assessed whether afferent and efferent populations were spatially distinct. To this end, the significant efferent and afferent Granger causality connections were visualized on spatial maps and we compared the individual geometric centres of each group using Hotelling’s T^2^ test for two multivariate dependent samples.

### Spiking activity

General measures of spiking activity such as firing rates and ratios between baseline and postural tremor were estimated. The timing of spikes relative to the background activity was assessed using the spike-related phase synchrony index (PSI, Lachaux *et al.*, 2002). The PSI was defined as:

(3) $$PSI_{t}=\;\frac{1}{N}\left|\overset{N}{\underset{n}{\sum }}\text{exp}(i\theta \left(t,n\right))\right|$$

where θ(t,n) is the phase difference of the trials at time t and N is the number of spikes. The segment of interest was defined as ±300 ms and in cases where there was high-frequency activity (multiple spikes within this window), only the first spike of the burst was considered. Significance was tested by randomly shuffling phase values 5000 times and re-estimating the PSI, producing a null distribution. This was done separately for background activity that significantly Granger caused EMG activity (efferent), for significant afferent Granger causality and where no causality existed. Additionally, phase interactions between pockets were analysed by estimating the average phase per subject at peak PSI, and examine the difference between in phases between afferent and efferent clusters.

### Data availability

The data that support the findings of this study are available from the corresponding author, upon reasonable request.

## Results

Across 12 hemispheres we obtained 41 recording trajectories over a total of 103 levels. Two patients contributed bilateral recordings, which were analysed separately. Independence between hemispheres is likely, not only because of laterality in tremor amplitude but also because of the differential effects of stimulation. For patient demographics see Table 1. On average, recording durations per electrode were 66.81 ± 4.40 s (postural tremor) and 65.01 ± 4.03 s (rest), while mean target coordinates for the *x*-, *y*- and *z*-axes were *x* = 11.5 ± 0.6 mm; *y* = − 18.9 ± 1.8 mm; *z* = − 0.4 ± 1.9 mm. A representation of all recordings is provided in Fig. 1, left column.

### Tremor-related power and coherence is spatially focal about ventral thalamus

Power at the individual tremor frequency increased at the more inferior parts of the ventrolateral thalamus. In a similar vein, coherence between the extensor digitorum communis muscle and contralateral recordings in the brain were likewise located in the lower parts of the thalamus (Fig. 1). The centre of maximal power was located at (11.5, −19.9, −0.8), while the coordinates for coherence maxima between spiking activity and EMG appeared slightly more ventral at (11.1, −19.5, −2.0). Nevertheless, peak power and coherence coordinates did not differ spatially according to Hotelling’s T^2^ test [T^2 ^= 4.17, *F*(3,7) = 1.08, *P* = 0.42]. Note that both tremor-related power and coherence are highly focal within the sampled region, ruling out a general effect of movement artefact. The Euclidean distances between maximal power and coherence coordinates and the determined active electrodes were 2.9 ± 1.1 mm and 3.0 ± 1.2, indicating a high agreement between both. Coherence between background activity (and EMG) and between spiking activity (and EMG) was also spatially inseparable [T^2 ^= 3.94, *F*(3,7) = 1.02, *P* = 0.44], as confirmed by permuting the peak coherence location (in 3D space) across subjects [5000 permutations, *F*(34 997) = 0.220, *P* = 0.883]. This was supported by a linear mixed-effects model, which demonstrated a significant relationship between coherence derived from background activity, and that derived from spiking activity [*F*(1356) = 6.07, *P* = 0.014; see Supplementary material]. Thus, background activity can be said to (at least partially) reflect direct neural engagement.

### Thalamic recordings reflect a mixed population of efferent and afferent activity

Using VAR models, we could identify significant afferent and efferent Granger causality between local background activity and tremulous EMG activity. The ratio of significant efferent connections to total connections was significantly higher than the ratio for afferent connections (Z = 2.57, *P* < 0.01, r = 0.27; *cf.*Fig. 2B). The importance of the central drive to tremor was additionally corroborated when we altered the model. Specifically, by creating a ‘virtual efferent lesion’ (setting the efferent VAR coefficients to zero offline) and re-estimating the tremor amplitude in the model, there was a consistent decrease of tremor amplitude. This result was consistent in all but one subject. In contrast, when undergoing a ‘virtual de-afferentation’ (that is, the VAR coefficients for feedback were set to zero offline), there was a heterogeneous change in tremor amplitude. Thus, effects between changes of efferent and afferent coefficients were significantly different, showing a consistent reduction in tremor amplitude in the efferent condition (Z = − 3.18, *P* < 0.001, r = − 0.33; Fig. 2C). Finally, we visualized the location of significant efferent and afferent Granger causality connections between EMG and microelectrode recordings (Fig. 2D). There was no significant difference between the geometric centres for significant efferent Granger causality connections (10.6, −20.3, −1.0) and afferent connections [10.8, −19.8, −1.1; Hotelling’s T^2 ^= 2.10, *F*(3,6) = 0.53, *P* = 0.68; Fig. 2D].

### Spiking rates predict changes in background activity and EMG tremor amplitude

**Figure 4:**
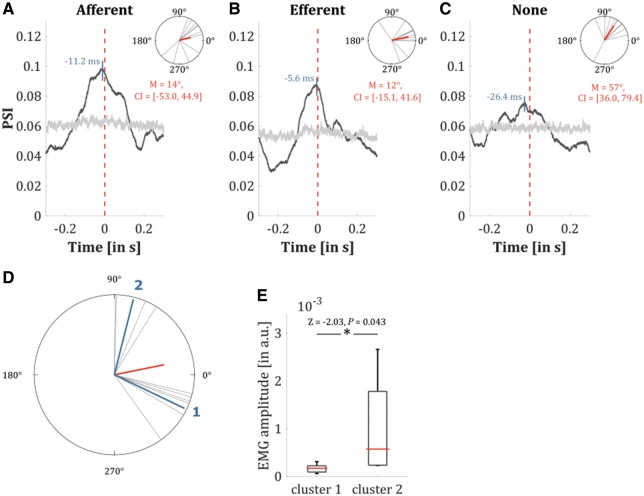
**PSI of the background activity when realigned to the occurrence of a spike at 0 ms (averaged over all hemispheres).** The maximum of PSI occurred at about −11 ms when only significant afferent Granger causality was selected (**A**) at −6 ms when only efferent connections were selected (**B**) and at about −26 ms when all connections were selected (**C**). The upper part shows the angles for every hemisphere at the individual maxima and the mean angle in red, whereas the grey line illustrates the significance level with permuted data (see main text). (**D**) The difference between angles at peak PSI for afferent and efferent connections for every hemisphere showing two distinct clusters (1 and 2) with phases at −25.6° (*n = *6) and 75.8° (*n = *4), respectively. (**E**) Tremor amplitude for subjects showing cluster 1 and cluster 2 were compared with a Rank sum test indicating a significantly higher amplitude when phase alignment between afferent and efferent connections peaked around 75.8° (*Z* = − 2.03, *P* = 0.043).

The change in firing rate compared to the baseline condition (rest) increased the closer the electrode was to the target. There was also a significant difference in the change in firing rate for those sites close to target versus further away (*Z* = − 2.02, *P* = 0.04, *r* = 0.41; Fig. 3C), indicating a direct relationship with the tremulous activity. On average, the firing rate ±2 mm from the target was 24.4 ± 8.6 s^−1^. With respect to spiking activity, the phase of the homogeneous background activity showed greatest synchronization around zero lag (Fig. 4A–C). Peak PSI values between spiking neurons with efferent and afferent connectivity on Granger causality and EMG were higher than the PSI for VLp-EMG combinations without significant Granger causality. Strikingly however, the differences in phase between afferent and efferent connections showed two separate clusters of activity (classified by *k*-means according to Lloyd, 1982) at −25.6° (*n = *6) and 75.8° (*n = *4), respectively (Fig. 4D). When comparing tremor amplitudes using the Rank sum test, there was significantly higher amplitude around the second (efferent) cluster, where the phase differences were 75.8° (Fig. 4E).

## Discussion

In this study, we have identified tremor-related power and thalamomuscular coherence increases relative to baseline that were spatially focal about ventral thalamus. By fitting causal models to the activity of cells we were able to classify pockets of background activity as predominantly efferent, predominantly afferent, or neither. We found that predominantly efferent pockets outnumbered afferent ones, but that their spatial topography was indistinguishable. This strongly supports an architecture where afferent and efferent populations co-exist in a mosaic of ‘tremor clusters’ (Pedrosa *et al.*, 2012). The importance of the efferent drive was underlined by virtual de-efferentation, which consistently diminished tremor amplitude, unlike de-afferentation. Resolvable spiking activity was phase-locked to background population spiking activity, most prominently when Granger causality for these pockets was significant. Furthermore, there is evidence that the phase-relationship between afferent and efferent populations may relate to the severity of tremor.

The importance of the thalamus in essential tremor is evident in the significant tremor relief attained by high-frequency DBS, particularly of the VLp and surrounding areas. Likewise, cerebellar infarctions and thalamotomy have been shown to attenuate essential tremor, and tremor-frequency remains unaffected through inertial manipulations that test the role of biomechanic feedback (Elble, 1986). Our results support the prominent role of the thalamus in this efferent tremor drive. In particular, we found that efferent Granger causality clusters significantly outnumbered afferent ones and that tremor amplitude in the EMG consistently diminished when supressing efferent drive in the VAR model. In contrast, after alterations of afferent connections, tremor amplitude remained, on average, unchanged. Using spectral Granger causality estimates, these results were shown to be frequency specific (Geweke, 1982; Dhamala *et al.*, 2008), relating directly to tremor activity. Nevertheless, there remained a strong representation of afferent activity within the thalamus. The latter may compromise macro-level coherence estimates with the peripheral tremor (Pedrosa *et al.*, 2014*b*).

We found that resolvable spiking activity was phase-locked to background population spiking activity at the electrode site. Phase synchronization was higher in connections where there was either significant efferent or afferent connectivity. Here we use the terms afferent and efferent connectivity to distinguish thalamic activity (indexed by spiking) that is better (Granger) predicted by EMG and better predicts EMG, respectively. The presumption is that thalamic activity that is better predicted by EMG is dominated by the effects of afferent input to the thalamus, whereas thalamic activity that better predicts the EMG is dominated by the effects of tremor driving inputs to the thalamus or intrinsic tremor driving mechanisms within the thalamus. Importantly, tremor amplitude appeared related to the phase difference between afferent and efferent connectivities, although this *post hoc* finding warrants further investigation.

The presented results are consistent with previous reports of different cells mediating efferent drive and afferent feedback (Lenz *et al.*, 1994). The lack of clear delay between the spiking activity and the background activity may be related to the mixing of efferent and afferent input in a small area. Yet the high representation of tremor related activity in more ventral recordings may be compatible with cerebellar input (Hirai and Jones, 1989; Hua and Lenz, 2005). Cerebellothalamic dysfunction underlying essential tremor has long been postulated and may parsimoniously explain many clinical signs encountered in essential tremor such as eye movement abnormalities, dysmetria or specific cognitive deficits such as verbal fluency reduction or disturbed time perception (Stoodley and Schmahmann, 2010; Pedrosa *et al.*, 2014*a*, 2016). Alternatively, one may speculate about cortico-thalamic or cortico-baso-thalamic contributions as there exist extensive reciprocal connections with the thalamus (Buzsaki and Draguhn, 2004), and these candidates have already been variously implicated in essential tremor (Raethjen and Deuschl, 2012, Pedrosa *et al.*, 2017).

The implication that the interplay of distinct thalamic cells mediate tremor amplitude in the periphery is an important one, since cortico-muscular (and thalamo-muscular) coherence could also merely reflect the presence of afferent feedback. Indeed, cortico-muscular coupling is often ambiguous in this regard (Hellwig *et al.*, 2000) and previous work has demonstrated strong afferent signals at tremor-frequency to be associated with ongoing tremor activity (Pedrosa *et al.*, 2014*b*). In another region, the subthalamic nucleus, tremor oscillations likewise suggested that 80% of the signal was attributed to sensory feedback, although this study involved parkinsonian patients (Tass *et al.*, 2010). Future studies may ascertain whether our results may be encountered in other tremor entities or distinct brain areas, despite the distinct characteristics of tremor in Parkinson’s disease and in essential tremor (di Biase *et al.* 2017). However, rather than dichotomize the functional roles of afferent and efferent connectivity, we speculate that it may be the integration of afferent and efferent signals within the VLp that is critical and determines tremor amplitude. This might be achieved, for example, through the competitive actions of afferent inputs and intrinsic or extrinsic tremor driving mechanisms upon oscillatory thalamic activity. This competitive action might involve phase dependent effects by analogy with the ability of thalamic stimulation to modulate tremor amplitude according to the phase at which it is delivered relative to the prevailing tremor oscillation (Cagnan *et al.*, 2017). Hence, in the current study, afferent and efferent connectivities were associated with different phases and these were, in turn, linked to differences in tremor amplitude.

In summary, we have identified the topography of neural populations that demonstrate increased activity and coherence with the peripheral tremor. The focality of this activity precludes the possibility that these results are due to movement artefacts in the recordings. We further isolated background activity from spiking populations and demonstrated that clusters of tremor-related activity can be classified as either predominantly efferent, predominantly afferent, or neither. These populations could not be topographically distinguished. Nevertheless, the specific alignment of phases between afferent and efferent subpopulations had a pronounced effect on tremor amplitude. By exploring the pathophysiological underpinnings of essential tremor, we lend considerable support to the notion that the thalamus is causally implicated in tremor generation.

## Supplementary Material

Supplementary Data

## Abbreviations

DBS: deep brain stimulation
PSI: phase synchrony index
VAR: vector-autoregressive model
VLp: posterior part of the ventrolateral thalamus
