# Supplementary material for: A functional micro-electrode mapping of ventral thalamus in essential tremor
Source: Brain. 2018 Jul 23;141(9):2644–54. doi: 10.1093/brain/awy192 (PMC6113647; doi:10.1093/brain/awy192)
Supplement: Supplementary Data [file awy192_suppl_data.pdf]

Supplementary material

Simulated data

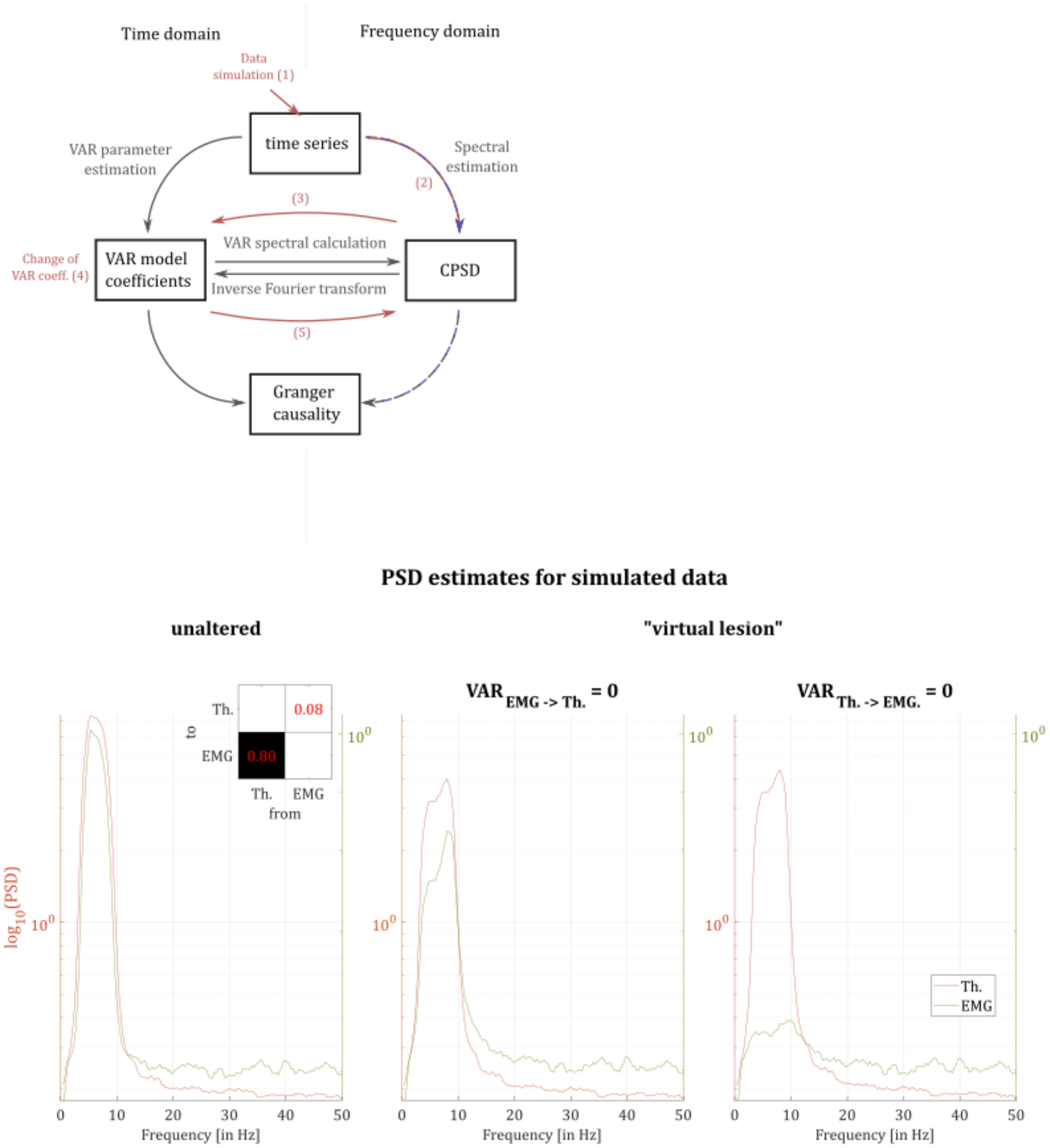

Supplementary Figure 1: On the upper part of the figure, a schematic exemplifies the different options to estimate Granger causality (GC). Time and frequency domain methods are thereby convertible (grey arrows). The blue arrow describes the steps applied to estimate GC in the manuscript. In the lower part, simulated data is shown. The same methods were used to validate the "virtual lesioning" detailed in the manuscript with all steps highlighted in the upper part of this figure in red. On the left, power spectra of two simulated signals (1) of 3 sec. duration and 250 repetitions with a strong 4-9 Hz component are shown. Similar to our data, the first time series (Th.) had a stronger GC effect on the second one (termed EMG; the estimates of frequency specific GC are shown on the right upper corner). After CPSD estimation (2), VAR

coefficients were determined (3) and either the Th.->EMG or the EMG->Th. Coefficient was set to zero (4). An inverse Fourier transform (5) resulted in the modified spectra of both signals which are illustrated on the right side. The results indicated that only the modification of the stronger (Th -> EMG, right plot) coefficient resulted in a modification of the second simulated time series' power spectrum.

Mixed-effects model

To assess whether coherence obtained between EMG and background activity relates directly to that obtained when considering spiking activity (i.e. do these measures [at least partially] represent a common neural signature), we constructed a linear mixed-effects model, shown below in Wilcoxon notation. The model is designed to test for a linear relationship in the strength of coherence between recording locations (within-subject), while accounting for differences in the gradient and intercept of that relationship between-subjects.

$$coh_{spike-EMG} \sim 1 + coh_{ba-emg} + (1 + coh_{ba-emg}|subject)$$

where $coh_{spike-emg}$ is the transformed (atanh) average coherence activity within the tremor-frequency range (+/- 3 Hz) between spiking activity and peripheral tremor; with coh_{ba-emg} the analogously defined transformed coherence between background activity and EMG.

The mixed-effects model provided a reasonable fit to the data (adjusted $R^2 = 0.21$) and revealed a significant main effect of coherence (between background activity and EMG) at tremor frequency ($F_{1,356} = 6.07$, $p = 0.014$). This indicates that, even when accounting for inter-individual differences, there was a significant relationship between spike-EMG coherence and background activity-EMG coherence within-subject between recording locations. Thus, background activity can be said to (at least partially) reflect direct neural engagement. Note that we have tested only a simple linear relationship here and have made no attempt to improve model performance by, for instance, considering only epochs with non-trivial levels of coherence.
